# Establishment of a matrix-assisted laser desorption/ionization time-of-flight mass spectrometry database and evaluation of different methods for cluster analysis of marine bacteria

**DOI:** 10.1099/jmm.0.002162

**Published:** 2026-05-08

**Authors:** Jia-Qi Guo, Jing Wu, He-Qing Cao, Yue Qiu, Xiao-Fei Liu

**Affiliations:** 1 School of Medical Laboratory, Shandong Second Medical University, Weifang, 261053, PR China; 2 Department of Laboratory Medicine, The 960th Hospital of the PLA Joint Logistics Support Force, Ji’nan, 250031, PR China

**Keywords:** 16S rDNA, *Aeromonas*, database, matrix-assisted laser desorption/ionization time-of-flight MS (MALDI-TOF MS), *rpoB*, *Vibrio*

## Abstract

**Introduction**. Accurate and rapid identification of marine bacteria is essential for the precise diagnosis and treatment of infectious diseases caused by marine pathogens. The accuracy of microbial identification using matrix-assisted laser desorption/ionization time-of-flight MS (MALDI-TOF MS) primarily depends on the diversity and number of strains included in the reference database.

**Hypothesis/Gap statement**. We hypothesized that expanding the MALDI-TOF MS database with a broader collection of protein spectral profiles from marine bacteria would significantly enhance identification accuracy for these strains.

**Aim**. This study aimed to establish a marine bacterial database to improve identification accuracy and to evaluate the applicability of MALDI-TOF MS-based cluster analysis for tracing the origin of clinical strains.

**Methodology**. We collected 203 strains isolated from marine environments and clinical samples, acquired their MALDI-TOF MS spectra and constructed a mass spectral database specific to marine bacteria. To validate the accuracy of the expanded database, 80 external strains were subsequently tested. Furthermore, we assessed the strain classification efficacy of MALDI-TOF MS cluster analysis and phylogenetic trees constructed from gene sequences.

**Results**. The species-level identification rate increased from 88.75 to 97.5%. The proportion of strains achieving a reliable identification score (>2.3) rose markedly from 43.75 to 91.25%. Cluster analysis based on MALDI-TOF MS demonstrated high accuracy in grouping bacteria at the species level. In addition, the maximum likelihood (ML) phylogenetic tree exhibited significantly higher bootstrap support values compared to the neighbour-joining tree.

**Conclusion**. The expanded marine bacterial database markedly enhances the accuracy and reliability of MALDI-TOF MS for identifying marine pathogens. For species identification and traceability, we recommend a combined strategy that includes initial MALDI-TOF MS screening and verification with phylogenetic trees based on the ML method.

## Data Summary

The individual accession number for the isolates can be found in Tables S1 and S2.

## Introduction

The marine environment harbours thousands of bacterial species, among which pathogens such as *Vibrio*, *Aeromonas* and *Salmonella* pose dual threats to aquaculture and human health [1,3]. As the most significant pathogenic group in marine environments, *Vibrio* species pose multiple infection risks [4]. For instance, *Vibrio vulnificus* infection can lead to septicaemia, with a mortality rate as high as 80% within 48 h [5,6], and *Vibrio parahaemolyticus* has become the leading cause of foodborne diseases along China’s coastal regions [7]. *Aeromonas* is another common marine pathogen that can enter the human body through wounds or contaminated food, secreting haemolysins and enterotoxins, leading to gastroenteritis and skin and soft-tissue infections [8]. Severe cases can progress to septicaemia [9]. Therefore, the accurate identification of marine bacteria is crucial.

Matrix-assisted laser desorption/ionization time-of-flight MS (MALDI-TOF MS) has become the mainstream method for clinical microbial identification, owing to its high efficiency and low cost [10,12]. In this technique, the mass spectra of bacteria in the test sample are compared with the spectra of known bacterial species in a reference database. Thus, the accuracy of bacterial identification largely depends on the diversity of strains in the reference database. Existing commercial databases have a limited number and variety of marine environmental isolates, leading to lower identification accuracy for marine bacterial species [13]. Studies have explored extending MS databases to improve bacterial identification accuracy [14]. In this study, we aimed to construct a marine bacterial database by integrating the mass spectra of marine environmental samples and clinical isolates, thereby expanding species coverage and improving species-level identification accuracy. Our database will provide technical support for the precise diagnosis of marine bacterial infections. Additionally, we performed systematic cluster analysis of MS data and gene sequencing data on dominant genera in our strain library, such as *Vibrio* and *Aeromonas*, in order to assess the classification performance of different analytical methods. Our findings will serve as a methodological foundation for future traceability studies of clinical strains.

## Methods

### Strains and cultivation

In this study, strains were obtained from two sources: environmental isolates and clinical isolates. Environmental isolates were obtained from the Marine Culture Collection of China and primarily originated in countries along the Pacific coast, including the USA, Japan, Australia and some regions of China. Clinical isolates were collected from the blood and wound exudates of infected patients at the 960th Hospital of the PLA Joint Logistics Support Force, Jinan, China. Detailed information on the strains in this study is provided in Table S1, available in the online Supplementary Material. All strains were cultured using a standardized procedure as described in the Manual of Clinical Microbiology. The strains were inoculated separately onto blood agar plates (Qingdao Hope Bio-Technology Co., Ltd., Qingdao, China) or marine agar 2216E culture media (Jinan Bio-Gene Technology Co., Ltd., Jinan, China) and incubated at 37 °C for 16–18 h.

### Gene sequencing analysis for species identification

Bacterial genomic DNA was extracted using the boiling method [15]. We amplified the *rpoB* gene of 117 *Vibrio* strains and the 16S rDNA gene of 86 bacterial strains belonging to different genera, including *Aeromonas*, *Pseudomonas*, *Brevundimonas*, *Aggregatibacterium*, *Edwardsiella*, *Photobacterium*, *Stenotrophomonas*, *Shewanella*, *Staphylococcus* and *Vagococcus*. The amplification products were verified using agarose gel electrophoresis and purified before being subjected to bidirectional sequencing using the Sanger method (BGI Genomics Co., Ltd., Shenzhen, China). The obtained gene sequences were compared with reference sequences in the National Center for Biotechnology Information database (http://www.ncbi.nlm.nih.gov/BLAST) using the blastn tool. The criteria for species identification were similarity ≥99% and coverage >95%.

### MALDI-TOF MS sample preparation

Fresh colonies were picked with an inoculation loop and placed in 300 µl of ultrapure water, to which 900 µl of pure ethanol was added. The tubes were vortexed for 1 min and centrifuged at 12,000 r.p.m. for 2 min. The supernatant was discarded, and the pellets were centrifuged again to remove ethanol residue and dried at room temperature. Then, the pellets were dissolved in 20 µl each of 70% formic acid and acetonitrile. The solution was mixed and centrifuged at 12,000 r.p.m. for 2 min. Subsequently, 1 µl of the supernatant was spotted onto MSP 96 polished steel target plates in eight replicates and overlaid with 1 µl *α*-cyano-4-hydroxycinnamic acid matrix solution (50% acetonitrile, 47.5% ultrapure water and 2.5% trifluoroacetic acid) after air-drying. After the sample was dried, it was placed into a Zybio EXS3000 mass spectrometer for analysis.

### Creation of main spectral projections using MALDI-TOF MS

We manually acquired linear MS data using Ex-Accuspec (*v*3.3.4.8) in positive ion mode (EXS3000 system, Zybio; mass range: 2–20 kDa). Bacterial samples were spotted onto eight target spots per isolate. Each spot was analysed thrice, with each analysis producing three spectra per spot. Thus, a total of 72 spectra were generated for each strain. As Zybio recommends, a bacterial test standard (*Escherichia coli* 25922) was used to calibrate the instrument before each acquisition session, ensuring that the average deviation of the molecular weight is less than 300 p.p.m. after correction. Low-quality spectra were excluded, and a main spectral projection (MSP) affiliated with each strain was created. The MSPs and corresponding strain information were incorporated into the MS database, enabling the sequential creation of a database encompassing all 203 isolates.

### Marine bacterial database evaluation

We randomly selected 80 strains (including 31 *Vibrio* strains), isolated from clinical patients and aquaculture animals, as validation strains. These strains were not included among the 203 strains in our self-built database. The validation strains were identified using gene sequencing. Each strain used for validation underwent sample preparation using the direct smear method. In brief, a single colony was evenly spread onto the target MALDI plate. After air-drying at room temperature, 1 µl of the matrix solution was applied onto the target plate, covering the sample. The sample was again allowed to air-dry at room temperature, and then the plate was loaded into the mass spectrometer for analysis. After the target plate was placed in the EXS3000 instrument, uniform white protein spots could be seen on the computer screen.

The combined database comprising both the self-built and commercial databases is defined as the expanded database. Two parameters were used to evaluate the accuracy of the expanded database: species-level identification and identification scores. First, the identification results were classified at the species and genus levels, and the identification percentage was calculated. Then, the identification scores were divided into three categories: >2.3, 2.0–2.3 and <2.0. Scores>2.3 indicate highly confident species-level identification. Scores of 2.0–2.3 indicate confident species-level identification, while scores<2.0 indicate confident genus-level identification only. The identification percentages for each category were calculated to assess the performance of the newly created database. Additionally, 31 *Vibrio* strains were used to specifically validate the expanded database for *Vibrio* species, and the results were evaluated based on the identification scores.

### Statistical analyses

The most abundant genera in our self-built database were *Vibrio* (117 strains) and *Aeromonas* (27 strains). We selected *Vibrio* isolates collected over the past decade to analyse interspecies phylogenetic relationships (36 strains). The *rpoB* gene sequences of the 36 *Vibrio* strains have been submitted to GenBank, and the accession numbers are provided in Table S1. Cluster analysis based on the MSPs was performed using EX-Smartspec software (*v*1.1.1.1001; Zybio Inc., Chongqing, China), employing cosine distance as the similarity metric and complete linkage as the agglomerative clustering method. Simultaneously, molecular phylogenetic trees based on *rpoB* gene sequences of *Vibrio* were constructed using MEGA *v*12.0 software and the maximum likelihood (ML) and neighbour-joining (NJ) methods. For the ML analysis, the Tamura–Nei model was applied as the nucleotide substitution model, with uniform rates among sites and employing 1,000 bootstrap replicates. Tree topology was optimized using the nearest-neighbour-interchange algorithm. For the NJ analysis, evolutionary distances were calculated using the *P*-distance model, also with 1,000 bootstrap replicates. Gaps and missing data were handled by partial deletion, and all codon positions were included in the analyses. Taxonomic differences between the two methods were assessed by comparing the topological structure of the phylogenetic trees. We also performed cluster analysis based on MSPs and constructed molecular phylogenetic trees based on the 16S rDNA gene using the ML and NJ methods for the second most abundant genus in our database: *Aeromonas*, isolated in the past decade (27 strains). We comprehensively analysed the classification results for the two bacterial genera to evaluate the classification capabilities of the different clustering methods. The 16S rRNA gene sequences of the 27 *Aeromonas* strains have been deposited in GenBank, and the accession numbers are provided in Table S2.

## Results

### Database development

To improve the identification accuracy of MALDI-TOF MS for marine-derived isolates, we obtained 203 bacterial strains from marine organisms and clinical patients to establish a bacterial protein fingerprinting database. Our self-built database covers 51 species from 11 genera, such as *Vibrio*, *Aeromonas* and *Shewanella*, as well as several rare genera. *Vibrio* (34 species) and *Aeromonas* (6 species) constitute the major components of the database. In contrast to the EXS3000 database *v*3.3.4.8, our database introduces five new *Vibrio* species (*Vibrio costicola*, *Vibrio fischeri*, *Vibrio logei*, *Vibrio carchariae*, *Vibrio atypicus*) and one new species of *Aeromonas* (*Aeromonas dhakensis*). The specific strains in our self-built database are shown in Table 1. Detailed information on the strains is provided in Table S3.

**Table 1.** Detailed strain counts in the newly created database

Species	Strain count	Species	Strain count
*Vibrio harveyi*	26	*Shewanella putrefaciens*	2
*Vibrio parahaemolyticus*	24	*Vibrio metschnikovii*	1
*Vibrio alginolyticus*	18	*Vibrio anguillarum*	1
*Pseudomonas aeruginosa*	15	*Vibrio natriegens*	1
*Brevundimonas aurantiaca*	11	*Vibrio aestuarianus*	1
*Aeromonas veronii*	10	*Vibrio costicola*	1
*Photobacterium damselae*	10	*Vibrio fischeri*	1
*Shewanella algae*	8	*Vibrio mediterranei*	1
*Edwardsiella tarda*	6	*Vibrio mimicus*	1
*Aeromonas media*	6	*Vibrio nereis*	1
*Vibrio vulnificus*	5	*Vibrio orientalis*	1
*Aeromonas hydrophila*	5	*Vibrio pelagia*	1
*Vibrio chagasii*	4	*Vibrio proteolyticus*	1
*Vibrio furnissii*	3	*Vibrio tubiashii*	1
*Stenotrophomonas maltophilia*	3	*Vibrio antiquarius*	1
*Aeromonas salmonicida*	3	*Vibrio logei*	1
*Vibrio fluvialis*	2	*Vibrio atypicus*	1
*Vibrio cholerae*	2	*Vibrio splendidus*	1
*Vibrio diazotrophicus*	2	*Vibrio rotiferianus*	1
*Vibrio campbellii*	2	*Vibrio neptunius*	1
*Vibrio cincinnatiensis*	2	*Staphylococcus aureus*	1
*Vibrio carchariae*	2	*Pseudomonas monteilii*	1
*Vibrio xuii*	2	*Aeromonas dhakensis*	1
*Vibrio fortis*	2	*Vagococcus fluvialis*	1
*Vibrio sinaloensis*	2	*Aggregatibacter actinomycetemcomitans*	1
*Aeromonas caviae*	2		

### Database evaluation

To validate the identification accuracy of our expanded database, we selected 80 strains for evaluation using both the EXS3000 mass spectrometer database *v*3.3.4.8 and expanded database. The identification results are shown in Table 2. Expanded database achieved a species-level identification accuracy of 97.5% (78/80), which was significantly higher than that of the commercial database (88.75%, 71/80). Neither database produced unreliable identification results. Further comparative analysis revealed that the expanded database achieved a high-confidence identification rate (score>2.3) of 91.25% (73/80), representing a significant increase over that of the commercial database (43.75%, 35/80; Table 3). Additionally, the rate of identification scores>2.3 for *Vibrio* species was 93.5% (29/31) in the expanded database and only 25/31 (80.65%) in the EXS3000 database (Table 4). Therefore, the combined use of our self-built database and the commercial database significantly improved the accuracy of the identification of marine environmental isolates.

**Table 2.** Assessment of the extended database at the species level

Level of identification	No. of identified strains/total strains	
	EXS3000 database *v*3.3.4.8	Expanded database
Species level	71/80 (88.75%)	78/80 (97.5%)
Genus level	9/80 (11.25%)	2/80 (2.5%)

**Table 3.** Evaluation of the extended database based on log scores

Log score	No. of identified strains/total strains	
	EXS3000 database *v*3.3.4.8	Expanded database
>2.3	35/80 (43.75%)	73/80 (91.25%)
2.0–2.3	41/80 (51.25%)	5/80 (6.25%)
<2.0	4/80 (5%)	2/80 (2.5%)

**Table 4.** Evaluation of the extended database based on identification scores for *Vibrio* genus

Log score	No. of identified strains/total strains	
	EXS3000 database V3.3.4.8	Expanded database
>2.3	25/31 (80.65%)	29/31 (93.5%)
2.0–2.3	5/31 (16.13%)	2/31 (6.4%)
<2.0	1/31 (3.23%)	0 (0%)

### MALDI-TOF MS cluster analysis and *rpoB* phylogenetic tree analysis of *Vibrio*

To analyse the intraspecific diversity of *Vibrio* species and evaluate classification performance between MSP-based cluster analysis and gene sequence-based phylogenetic trees, we performed phylogenetic analysis on 36 *Vibrio* strains.

**Fig. 1.**
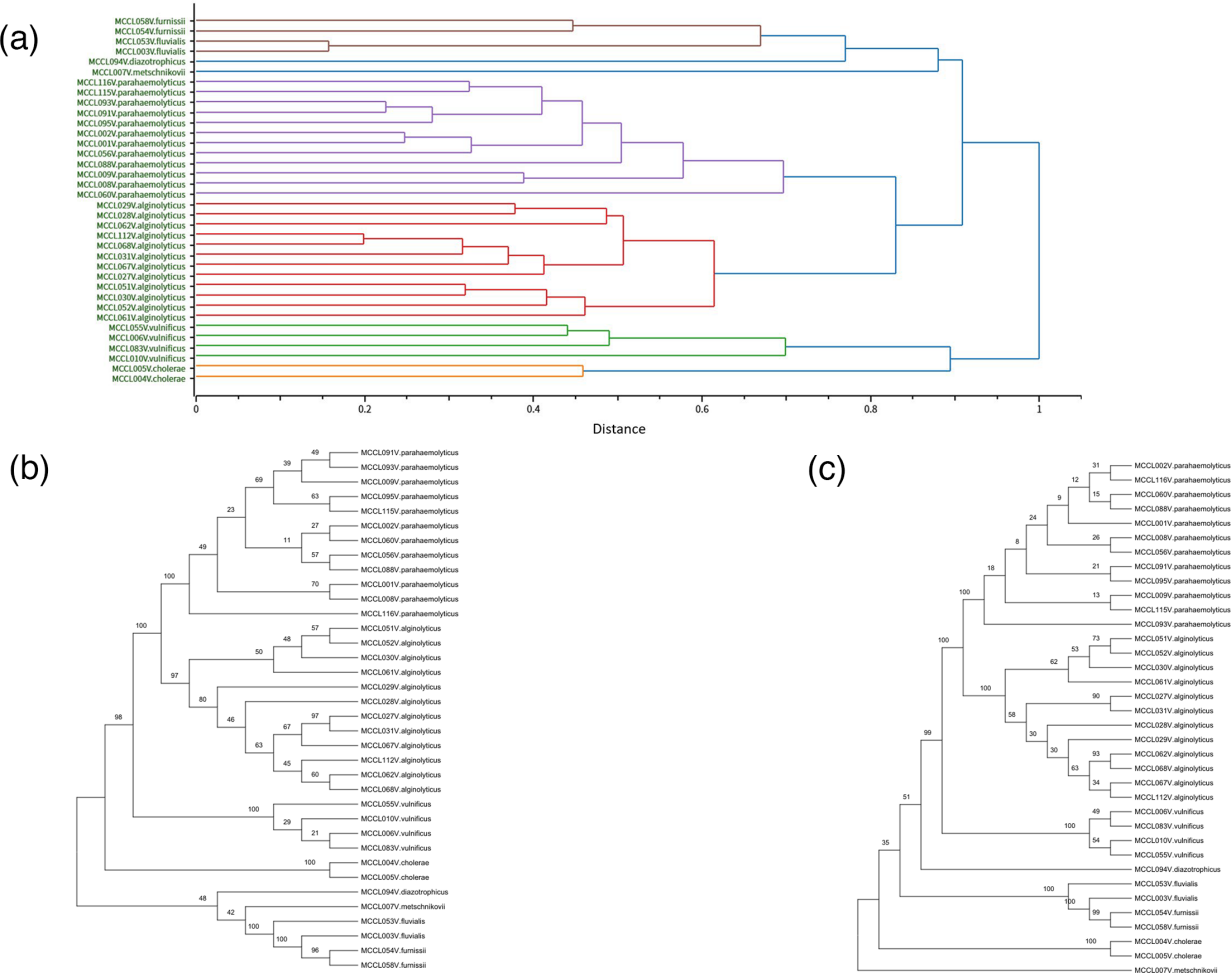
(**a**) MSP tree diagram based on 36 *Vibrio* strains in the extended database. (**b**) ML phylogenetic tree based on *rpoB* sequences of 36 *Vibrio* strains. The robustness of the branching is indicated by bootstrap values calculated for 1,000 subsets. (**c**) Neighbour linkage (NJ) phylogenetic tree based on *rpoB* sequences of 36 *Vibrio* strains. The robustness of the branching is indicated by bootstrap values calculated for 1,000 subsets.

Clustering analysis based on MSPs successfully clustered *Vibrio* species into distinct monophyletic groups, revealing distinct branches that signify substantial inter-strain differences. The clustering of *V. parahaemolyticus* and *Vibrio alginolyticus* at a high similarity of 0.83, pointing to a close relationship in their protein compositions (Fig. 1a). Although the phylogenetic trees generated by the ML and NJ methods differ in topological details, both approaches accurately classified all *Vibrio* strains at the species level, as shown in Fig. 1(b, c). Notably, the ML tree showed substantially higher bootstrap support values at a majority of internal nodes compared to the NJ tree.

### MALDI-TOF MS cluster analysis and 16S rDNA phylogenetic tree analysis of *Aeromonas*

**Fig. 2.**
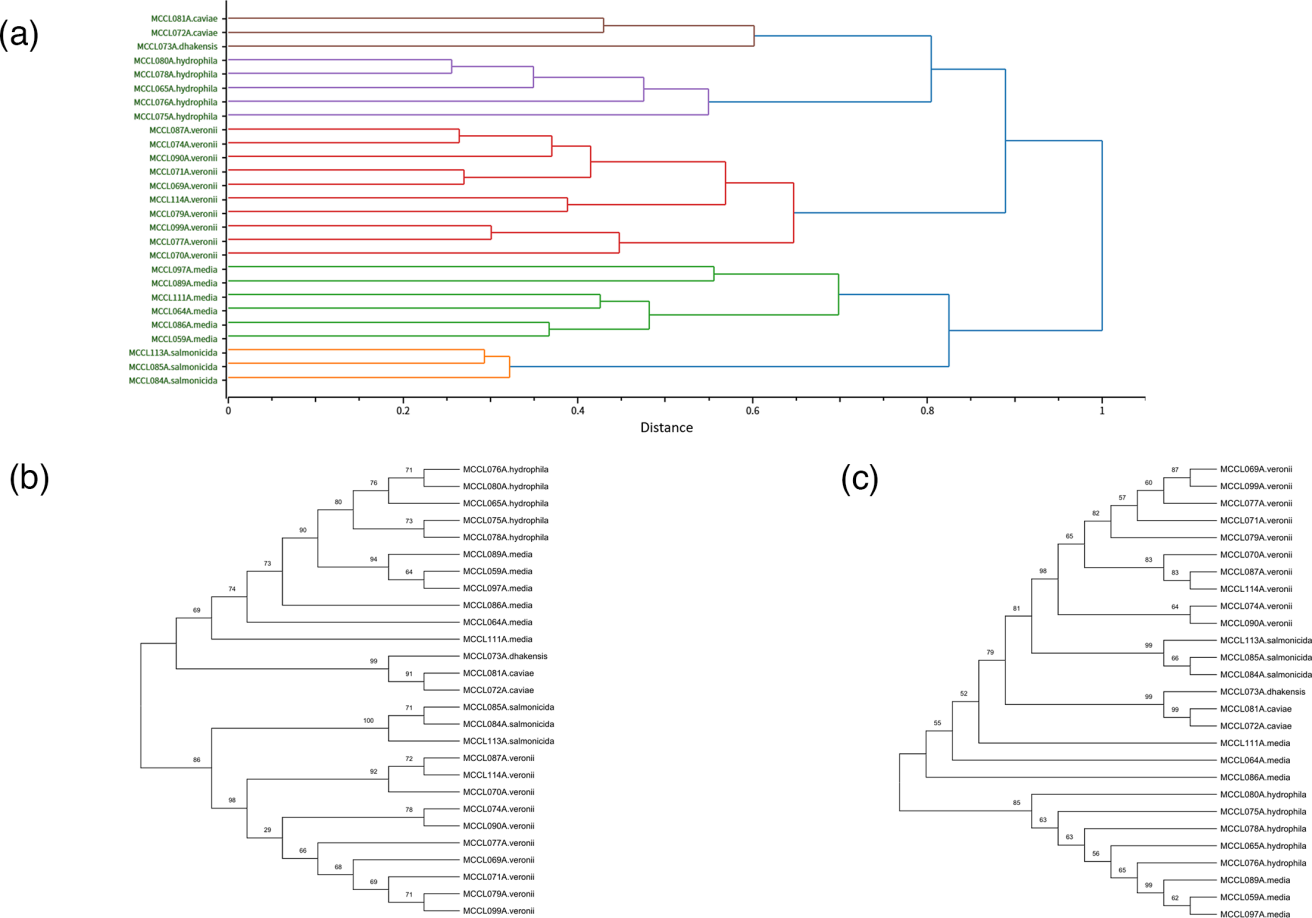
(**a**) MSP tree diagram based on 27 *Aeromonas* species in the extended database. (**b**) ML phylogenetic tree based on 16S rDNA sequences of 27 *Aeromonas* species. The robustness of the branching is indicated by bootstrap values calculated for 1,000 subsets. (**c**) Neighbour linkage (NJ) phylogenetic tree based on 16S rDNA sequences of 27 *Aeromonas* species. The robustness of the branching is indicated by bootstrap values calculated for 1,000 subsets.

We selected 27 *Aeromonas* strains to analyse the intraspecific diversity of *Aeromonas* species and evaluate differences in classification performance between MSP-based cluster analysis and 16S rDNA gene sequence-based phylogenetic trees. The cluster analysis topology is shown in Fig. 2(a). *Aeromonas caviae*, *A*. *dhakensis* and *Aeromonas hydrophila* clustered together at a distance of 0.85 on the dendrogram (Cluster I), and *Aeromonas media* and *Aeromonas salmonicida* formed adjacent branches (Cluster II). In contrast, *Aeromonas veronii* formed a separate evolutionary branch (Cluster III).

As shown in Fig. 2(b), the ML tree indicates that *A. media* and *A. hydrophila* cluster together on the same branch, with high bootstrap support, suggesting a close evolutionary relationship between the two species. In contrast, the NJ tree (Fig. 2c) displays a different topological structure: *A. media* does not cluster closely with *A. hydrophila*, and its phylogenetic placement is markedly altered. Additionally, the bootstrap support values in the ML tree are generally higher than those in the NJ tree.

## Discussion

Many authors have supplemented commercial databases with self-built MS databases to enhance microbial identification accuracy. For instance, Garrigos *et al.* established an *Achromobacter* genus database based on the Bruker platform and achieved correct identification rates of 99.4% (166/167) at identification scores≥2.0; in contrast, the Bruker commercial database only identified 50.9% (85/167) of the species accurately [14]. Lasch *et al.* developed a database of highly pathogenic bacteria to improve clinical identification accuracy [16]. Thus far, studies have focused on expanding databases with terrestrial strains [17,19], and databases constructed using marine strains are relatively rare. Our self-built database consists entirely of strains obtained from marine environments and clinical settings. Additionally, most studies that have built microbial databases using MALDI-TOF MS have used the Bruker MS platform [20,22], whereas we used the Zybio EXS3000 mass spectrometer in our study. Since mass spectrometers from different brands do not work well together, building a local database with our current mass spectrometer will help identify strains in our future clinical work. Our self-built marine bacterial MS database complements the EXS3000 database (*v*3.3.4.8) and includes strains from China, the USA, Australia, Thailand, Japan and more. Compared to the original database, it includes five new species of *Vibrio* and one new species of *Aeromonas*. We evaluated the identification accuracy of our expanded database. The results showed that our database significantly improved the identification accuracy for marine environmental isolates. The substantial increase in identification accuracy (>2.3), from 43.75 to 91.25%, may be due to 41% of the strains being collected from aquatic organisms in Shandong Province or isolated from local clinical patients. The validation strains are closely related to some strains used to build the database. Moreover, most patients had diseases caused by infections in the local area, and these infecting strains are closely related to the strains in the self-built database. Therefore, including such strains in the MS database can boost identification accuracy. In the future, we will continue to expand the number and types of marine bacteria, collect more marine pathogenic bacteria and incorporate them into the MS database, thereby improving the accuracy of identification.

Marine bacterial tracing can identify the sources and transmission pathways of infections, thereby effectively controlling outbreaks [23]. During foodborne disease outbreaks, tracing can rapidly identify contamination sources and specific pathogenic strains, enabling swift interruption of transmission chains to contain the epidemic. Tracing can also predict the evolutionary trends of pathogenic bacteria [24,25]. Fearnley *et al.* conducted a traceability investigation into the 2021–2022 national *V. parahaemolyticus* enteritis outbreak in Australia, identifying the source of infection as Australian native oysters. They identified the pathogens as two specific strains, ST417 and ST50. Through strain traceability, the spread of the outbreak was effectively contained [26]. Cluster analysis is a method for strain tracing and can be performed using gene sequences as well as MSPs. At the gene level, studies [27,28] have mainly used 16S rDNA and *rpoB* gene sequencing for *Vibrio* species identification as well as for phylogenetic analysis. However, the *rpoB* gene provides approximately three times the resolution of the 16S rDNA gene for *Vibrio* species [29]. Given the potential lack of sequence conservation of the 16S rDNA gene within the *Vibrio* genus, we constructed a phylogenetic tree based on the *rpoB* gene sequences of 36 *Vibrio* strains. A corresponding phylogenetic tree was also constructed from 16S rDNA sequences of 27 *Aeromonas* strains. The results showed that phylogenetic trees generated using the ML and NJ methods exhibited highly congruent classification patterns at the species level. However, the ML tree demonstrated significantly higher bootstrap support values at nodes, particularly for closely related species such as *V. parahaemolyticus* and *V. alginolyticus*. These topological discrepancies may be attributed to fundamental differences between the two algorithms: the NJ method, which relies on linear pairwise genetic distance calculations, is more sensitive to homologous recombination or localized mutations in the sequences; in contrast, the ML method optimizes evolutionary pathways using a probabilistic substitution model, providing greater global consistency and statistical robustness [30]. Additionally, cluster analysis based on MALDI-TOF MS also enabled accurate strain grouping at the species level. However, MALDI-TOF MS primarily reflects high-abundance protein profiles in bacteria, whereas gene sequence analysis is more sensitive for detecting subtle genetic variations [31].

Genome sequencing and MALDI-TOF MS are commonly used for microbial identification and cluster analysis. Genome sequencing remains the ‘gold standard’ for bacterial identification [32], but it is time-consuming and costly. Moreover, the sequencing of a single gene cannot fully represent all of the genomic information of the microbes. Therefore, it is essential to consider multi-gene sequencing to enhance the reliability of the results [33,34]. In contrast, identification and cluster analysis based on MS profiles are faster, simpler and more practical, making them more suitable for clinical applications. Therefore, in clinical or environmental monitoring, MALDI-TOF MS can be used for the preliminary identification and analysis of bacteria, and molecular validation may be applied to strains with uncertain MS classification results to improve typing accuracy.

In summary, a self-built MALDI-TOF MS database can enhance laboratory identification of marine bacteria, particularly for bacterial strains that are scarce in commercial databases or difficult to locate using morphological or biochemical methods. Therefore, laboratories should collect bacterial strain samples from their regions and establish an expanded database to improve the accuracy of bacterial identification. Additionally, we will expand the diversity and quantity of bacterial strains in our self-built database and optimize cluster analysis algorithms to improve the accuracy and practicality of the database. In clinical settings, combining MS with gene sequencing can enhance the timeliness and accuracy of bacterial identification. Incorporating more marine bacteria into our MALDI-TOF database enables rapid, low-cost preliminary tracing through MSP clustering during clinical outbreaks of bacterial infections. This provides a basis for determining infection sources and guiding rational drug use. While MALDI-TOF-based clustering analysis cannot replace precise genomic-level localization, it holds significant early warning value and public health significance.

## Supplementary material

10.1099/jmm.0.002162 Uncited Supplementary Material 1.

## Abbreviations

MALDI-TOF MS: matrix-assisted laser desorption/ionization time-of-flight MS
ML: maximum likelihood
MSP: main spectral projection
NJ: neighbour-joining
