# Supplementary material for: Establishment of a matrix-assisted laser desorption/ionization time-of-flight mass spectrometry database and evaluation of different methods for cluster analysis of marine bacteria
Source: J Med Microbiol. 2026 May 8;75(5):002162. doi: 10.1099/jmm.0.002162 (PMC13155701; doi:10.1099/jmm.0.002162)
Supplement: Uncited Supplementary Material 1. [file jmm-75-02162-s001.pdf]

Supplementary Table 1. GenBank accession numbers of the *Vibrio* strains used for cluster analysis.

Self-built database number	Strain	GenBank ID
MCCL001	*Vibrio parahaemolyticus*	PX436196
MCCL002	*Vibrio parahaemolyticus*	PX436197
MCCL003	*Vibrio fluvialis*	PX436198
MCCL004	*Vibrio cholerae*	PX436199
MCCL005	*Vibrio cholerae*	PX436200
MCCL006	*Vibrio vulnificus*	PX436201
MCCL007	*Vibrio metschnikovii*	PX436202
MCCL008	*Vibrio parahaemolyticus*	PX436203
MCCL009	*Vibrio parahaemolyticus*	PX436204
MCCL010	*Vibrio vulnificus*	PX436205
MCCL027	*Vibrio alginolyticus*	PX436206
MCCL028	*Vibrio alginolyticus*	PX436207
MCCL029	*Vibrio alginolyticus*	PX436208
MCCL030	*Vibrio alginolyticus*	PX436209
MCCL031	*Vibrio alginolyticus*	PX436210
MCCL051	*Vibrio alginolyticus*	PX436211
MCCL052	*Vibrio alginolyticus*	PX436212
MCCL053	*Vibrio fluvialis*	PX436213
MCCL054	*Vibrio furnissii*	PX436214
MCCL055	*Vibrio vulnificus*	PX436215
MCCL056	*Vibrio parahaemolyticus*	PX436216
MCCL058	*Vibrio furnissii*	PX436217
MCCL060	*Vibrio parahaemolyticus*	PX436218
MCCL061	*Vibrio alginolyticus*	PX436219
MCCL062	*Vibrio alginolyticus*	PX436220
MCCL067	*Vibrio alginolyticus*	PX436221
MCCL068	*Vibrio alginolyticus*	PX436222
MCCL083	*Vibrio vulnificus*	PX436223
MCCL088	*Vibrio parahaemolyticus*	PX436224
MCCL091	*Vibrio parahaemolyticus*	PX436225
MCCL093	*Vibrio parahaemolyticus*	PX436226
MCCL094	*Vibrio diazotrophicus*	PX436227
MCCL095	*Vibrio parahaemolyticus*	PX436228
MCCL112	*Vibrio alginolyticus*	PX436229
MCCL 115	*Vibrio parahaemolyticus*	PX436230
MCCL 116	*Vibrio parahaemolyticus*	PX436231

Supplementary Table 2. GenBank accession numbers of the *Aeromonas* strains used for cluster analysis.

Self-built database number	Strain	GenBank ID
MCCL059	*Aeromonas media*	PX426651
MCCL064	*Aeromonas media*	PX426652
MCCL065	*Aeromonas hydrophila*	PX426653
MCCL069	*Aeromonas veronii*	PX426654
MCCL070	*Aeromonas veronii*	PX426655
MCCL071	*Aeromonas veronii*	PX426656
MCCL072	*Aeromonas caviae*	PX426657
MCCL073	*Aeromonas dhakensis*	PX426658
MCCL074	*Aeromonas veronii*	PX426659
MCCL075	*Aeromonas hydrophila*	PX426660
MCCL076	*Aeromonas hydrophila*	PX426661
MCCL077	*Aeromonas veronii*	PX426662
MCCL078	*Aeromonas hydrophila*	PX426663
MCCL079	*Aeromonas veronii*	PX426664
MCCL080	*Aeromonas hydrophila*	PX426665
MCCL081	*Aeromonas caviae*	PX426666
MCCL084	*Aeromonas salmonicida*	PX426667
MCCL085	*Aeromonas salmonicida*	PX426668
MCCL086	*Aeromonas media*	PX426669
MCCL087	*Aeromonas veronii*	PX426670
MCCL089	*Aeromonas media*	PX426671
MCCL090	*Aeromonas veronii*	PX426672
MCCL097	*Aeromonas media*	PX426673
MCCL099	*Aeromonas veronii*	PX426674
MCCL111	*Aeromonas media*	PX426675
MCCL113	*Aeromonas salmonicida*	PX426676
MCCL114	*Aeromonas veronii*	PX426677

Supplementary Table 3. Information on Bacterial Strains Used for the Construction of the MALDI-TOF MS Database.

Self-built database number	Strain name	Collection location	Collection Time	Separation substrate
MCCL001	*vibrio parahaemolyticus*	Jinan, Shandong, China	2017	Human, Stool
MCCL002	*vibrio parahaemolyticus*	Jinan, Shandong, China	2019	Human, Stool
MCCL003	*vibrio fluvialis*	Jinan, Shandong, China	2019	Human, Blood
MCCL004	*vibrio cholerae*	Jinan, Shandong, China	2022	Human, Blood
MCCL005	*vibrio cholerae*	Jinan, Shandong, China	2023	Human, Blood
MCCL006	*Vibrio vulnificus*	Jinan, Shandong, China	2024	Human, Blood
MCCL007	*Vibrio metschnikovii*	Jinan, Shandong, China	2024	Human, Blood
MCCL008	*Vibrio parahaemolyticus*	Jinan, Shandong, China	2024	Human, Wound Drainage
MCCL009	*Vibrio parahaemolyticus*	Jinan, Shandong, China	2023	Human, Blood
MCCL010	*Vibrio vulnificus*	Jinan, Shandong, China	2024	Human, Wound Drainage
MCCL011	*Edwardsiella tarda*	Qingdao, Shandong, China	2006	turbot
MCCL012	*Edwardsiella tarda*	Qingdao, Shandong, China	2006	turbot
MCCL013	*Edwardsiella tarda*	Qingdao, Shandong, China	2006	turbot
MCCL014	*Edwardsiella tarda*	Qingdao, Shandong, China	2006	turbot
MCCL015	*Edwardsiella tarda*	Qingdao, Shandong, China	2006	turbot
MCCL016	*Brevundimonas aurantiaca*	Qingdao, Shandong, China	2008	Seawater
MCCL017	*Brevundimonas aurantiaca*	Qingdao, Shandong, China	2008	Seawater
MCCL018	*Brevundimonas aurantiaca*	Qingdao, Shandong, China	2008	Seawater
MCCL019	*Brevundimonas aurantiaca*	Qingdao, Shandong, China	2008	Seawater
MCCL020	*Brevundimonas aurantiaca*	Qingdao, Shandong, China	2008	Seawater
MCCL021	*Brevundimonas aurantiaca*	Qingdao, Shandong, China	2008	Seawater
MCCL022	*Brevundimonas aurantiaca*	Qingdao, Shandong, China	2008	Seawater
MCCL023	*Brevundimonas aurantiaca*	Qingdao, Shandong, China	2008	Seawater

MCCL024	*Brevundimonas aurantiaca*	Qingdao, Shandong, China	2008	Seawater
MCCL025	*Brevundimonas aurantiaca*	Qingdao, Shandong, China	2008	Seawater
MCCL026	*Brevundimonas aurantiaca*	Qingdao, Shandong, China	2008	Seawater
MCCL027	*Vibrio alginolyticus*	Rizhao, Shandong, China	2016	Seawater
MCCL028	*Vibrio alginolyticus*	Rizhao, Shandong, China	2016	Seawater
MCCL029	*Vibrio alginolyticus*	Rizhao, Shandong, China	2016	Seawater
MCCL030	*Vibrio alginolyticus*	Rizhao, Shandong, China	2016	Seawater
MCCL031	*Vibrio alginolyticus*	Rizhao, Shandong, China	2016	Seawater
MCCL032	*Pseudomonas aeruginosa*	Qingdao, Shandong, China	2008	Seawater
MCCL033	*Stenotrophomonas maltophilia*	Qingdao, Shandong, China	2008	Seawater
MCCL034	*Stenotrophomonas maltophilia*	Qingdao, Shandong, China	2008	Seawater
MCCL035	*Staphylococcus aureus*	Qingdao, Shandong, China	2007	shrimp
MCCL036	*Pseudomonas aeruginosa*	Qingdao, Shandong, China	2008	Seawater
MCCL037	*Pseudomonas aeruginosa*	Qingdao, Shandong, China	2008	Seawater
MCCL038	*Pseudomonas aeruginosa*	Qingdao, Shandong, China	2008	Seawater
MCCL039	*Pseudomonas aeruginosa*	Qingdao, Shandong, China	2008	Seawater
MCCL040	*Pseudomonas aeruginosa*	Qingdao, Shandong, China	2008	Seawater
MCCL041	*Pseudomonas aeruginosa*	Qingdao, Shandong, China	2008	Seawater
MCCL042	*Pseudomonas aeruginosa*	Qingdao, Shandong, China	2008	Seawater
MCCL043	*Pseudomonas aeruginosa*	Qingdao, Shandong, China	2008	Seawater
MCCL044	*Pseudomonas aeruginosa*	Qingdao, Shandong, China	2008	Seawater
MCCL045	*Pseudomonas aeruginosa*	Qingdao, Shandong, China	2008	Seawater
MCCL046	*Pseudomonas aeruginosa*	Qingdao, Shandong, China	2008	Seawater
MCCL047	*Pseudomonas aeruginosa*	Qingdao, Shandong, China	2008	Seawater

MCCL048	*Pseudomonas aeruginosa*	Qingdao, Shandong, China	2008	Seawater
MCCL049	*Pseudomonas aeruginosa*	Qingdao, Shandong, China	2008	Seawater
MCCL050	*Stenotrophomonas maltophilia*	Qingdao, Shandong, China	2008	Seawater
MCCL051	*Vibrio alginolyticus*	Weihai, Shandong, China	2018	Seawater
MCCL052	*Vibrio alginolyticus*	Weihai, Shandong, China	2018	Seawater
MCCL053	*Vibrio fluvialis*	Weihai, Shandong, China	2018	Seawater
MCCL054	*Vibrio furnissii*	Weihai, Shandong, China	2018	Seawater
MCCL055	*Vibrio vulnificus*	Weihai, Shandong, China	2018	Seawater
MCCL056	*Vibrio parahaemolyticus*	Weihai, Shandong, China	2018	Seawater
MCCL057	*Pseudomonas monteilii*	Weihai, Shandong, China	2018	Seawater
MCCL058	*Vibrio furnissii*	Weihai, Shandong, China	2018	Seawater
MCCL059	*Aeromonas media*	Jinan, Shandong, China	2024	Seawater
MCCL060	*Vibrio parahaemolyticus*	Jinan, Shandong, China	2024	Seawater
MCCL061	*Vibrio alginolyticus*	Weihai, Shandong, China	2018	Human, wound exudate
MCCL062	*Vibrio alginolyticus*	Weihai, Shandong, China	2018	Seawater
MCCL063	*Photobacterium damselae*	Jinan, Shandong, China	2024	Seawater
MCCL064	*Aeromonas media*	Jinan, Shandong, China	2024	shrimp
MCCL065	*Aeromonas hydrophila*	Jinan, Shandong, China	2024	Seawater
MCCL066	*Shewanella algae*	Jinan, Shandong, China	2024	shrimp
MCCL067	*Vibrio alginolyticus*	Weihai, Shandong, China	2018	Seawater
MCCL068	*Vibrio alginolyticus*	Weihai, Shandong, China	2018	Seawater
MCCL069	*Aeromonas veronii*	Jinan, Shandong, China	2024	Seawater
MCCL070	*Aeromonas veronii*	Jinan, Shandong, China	2024	Seawater
MCCL071	*Aeromonas veronii*	Jinan, Shandong, China	2024	Seawater
MCCL072	*Aeromonas caviae*	Jinan, Shandong, China	2024	Seawater
MCCL073	*Aeromonas dhakensis*	Jinan, Shandong, China	2024	Seawater
MCCL074	*Aeromonas veronii*	Jinan, Shandong, China	2024	Seawater
MCCL075	*Aeromonas hydrophila*	Jinan, Shandong, China	2024	Seawater
MCCL076	*Aeromonas hydrophila*	Jinan, Shandong, China	2024	Seawater
MCCL077	*Aeromonas veronii*	Jinan, Shandong, China	2024	Seawater
MCCL078	*Aeromonas hydrophila*	Jinan, Shandong, China	2024	Seawater
MCCL079	*Aeromonas veronii*	Jinan, Shandong, China	2024	Seawater
MCCL080	*Aeromonas hydrophila*	Jinan, Shandong, China	2024	Seawater
MCCL081	*Aeromonas caviae*	Jinan, Shandong, China	2024	Seawater
MCCL082	*Vagococcus fluvialis*	Jinan, Shandong, China	2024	Seawater
MCCL083	*Vibrio vulnificus*	Jinan, Shandong, China	2024	shrimp
MCCL084	*Aeromonas salmonicida*	Jinan, Shandong, China	2024	Seawater
MCCL085	*Aeromonas salmonicida*	Jinan, Shandong, China	2024	Seawater
MCCL086	*Aeromonas media*	Jinan, Shandong, China	2024	Seawater

MCCL087	*Aeromonas veronii*	Jinan, Shandong, China	2024	Seawater
MCCL088	*Vibrio parahaemolyticus*	Jinan, Shandong, China	2024	shrimp
MCCL089	*Aeromonas media*	Jinan, Shandong, China	2024	Seawater
MCCL090	*Aeromonas veronii*	Jinan, Shandong, China	2024	Seawater
MCCL091	*Vibrio parahaemolyticus*	Jinan, Shandong, China	2024	Seawater
MCCL092	*Photobacterium damselae*	Jinan, Shandong, China	2024	Seawater
MCCL093	*Vibrio parahaemolyticus*	Jinan, Shandong, China	2024	Seawater
MCCL094	*Vibrio diazotrophicus*	Jinan, Shandong, China	2024	Seawater
MCCL095	*Vibrio parahaemolyticus*	Jinan, Shandong, China	2024	Seawater
MCCL096	*Photobacterium damselae*	Jinan, Shandong, China	2024	Seawater
MCCL097	*Aeromonas media*	Jinan, Shandong, China	2024	Seawater
MCCL098	*Shewanella putrefaciens*	Jinan, Shandong, China	2024	Seawater
MCCL099	*Aeromonas veronii*	Jinan, Shandong, China	2024	Seawater
MCCL100	*Photobacterium damselae*	Jinan, Shandong, China	2024	Seawater
MCCL101	*Shewanella algae*	Jinan, Shandong, China	2024	Seawater
MCCL102	*Edwardsiella tarda*	Jinan, Shandong, China	2016	Seawater
MCCL103	*Shewanella algae*	Jinan, Shandong, China	2025	Seawater
MCCL104	*Shewanella algae*	Jinan, Shandong, China	2025	Seawater
MCCL105	*Shewanella algae*	Jinan, Shandong, China	2025	Seawater
MCCL106	*Shewanella algae*	Jinan, Shandong, China	2025	Seawater
MCCL107	*Shewanella algae*	Jinan, Shandong, China	2025	Seawater
MCCL108	*Shewanella putrefaciens*	Jinan, Shandong, China	2025	Seawater
MCCL109	*Shewanella algae*	Jinan, Shandong, China	2025	Seawater
MCCL110	*Photobacterium damselae*	Jinan, Shandong, China	2025	Seawater
MCCL111	*Aeromonas media*	Jinan, Shandong, China	2025	Seawater
MCCL112	*Vibrio alginolyticus*	Jinan, Shandong, China	2024	Seawater
MCCL113	*Aeromonas salmonicida*	Jinan, Shandong, China	2025	Seawater
MCCL114	*Aeromonas veronii*	Jinan, Shandong, China	2025	Seawater
MCCL115	*Vibrio parahaemolyticus*	Jinan, Shandong, China	2025	Seawater
MCCL116	*Vibrio parahaemolyticus*	Jinan, Shandong, China	2025	Seawater
MCCL117	*Photobacterium damselae*	Jinan, Shandong, China	2025	Seawater
MCCL118	*Vibrio anguillarum*	fjords Norway	1909	cod
MCCL119	*Vibrio carchariae*	Hawaii United States	1991	seawater
MCCL120	*Vibrio damsela*	USA	1981	Damsel fish
MCCL121	*Vibrio diazotrophicus*	Nova Scotia Canada	1981	sea urchin
MCCL122	*Vibrio furnissii*	Japan	1983	human, faeces
MCCL123	*Vibrio natriegens*	USA	1982	oyster
MCCL124	*Vibrio vulnificus*	USA	1979	human, blood
MCCL125	*Vibrio harveyi*	Thailand	1990	Shrimp

MCCL126	*Vibrio parahaemolyticus*	Thailand	2006	shrimp
MCCL127	*Vibrio parahaemolyticus*	Thailand	2006	shrimp
MCCL128	*Vibrio parahaemolyticus*	Thailand	2006	shrimp
MCCL129	*Vibrio parahaemolyticus*	Thailand	2006	shrimp
MCCL130	*Vibrio harveyi*	Qingdao, Shandong, China	2007	shrimp
MCCL131	*Vibrio harveyi*	Qingdao, Shandong, China	2007	shrimp
MCCL132	*Vibrio aestuarianus*	USA	1983	Oyster
MCCL133	*Vibrio alginolyticus*	Japan	1961	horse mackerel
MCCL134	*Vibrio alginolyticus*	Japan	1961	horse mackerel
MCCL135	*Vibrio harveyi*	USA	1936	dead amphipod
MCCL136	*Vibrio cincinnatiensisi*	Cincinnati Hospital	1986	Human, Blood and cerebrospinal fluid
MCCL137	*Vibrio costicola*	Australian	1938	Bacon curing brine
MCCL138	*Vibrio fisheri*	China	1889	Seawater
MCCL139	*Vibrio mediterranei*	Valencia Spain	1986	coastal marine plankton
MCCL140	*Vibrio mimicus*	USA	1981	human, ear
MCCL141	*Vibrio nereis*	USA	1973	seawater
MCCL142	*Vibrio orientalis*	Yellow Sea	1983	seawater
MCCL143	*Vibrio parahaemolyticus*	Japan	1951	Human, Stool
MCCL144	*Vibrio pelagia*	USA	1971	seawater
MCCL145	*Vibrio proteolyticus*	USA	1964	wood-boring isopod
MCCL146	*Vibrio tubiashii*	USA	1984	hard clam
MCCL147	*Vibrio sinaloensis*	Yellow Sea, China	2008	Seawater
MCCL148	*Vibrio logei*	USA	1964	Pacific cod
MCCL149	*Vibrio carchariae*	Bahamas	2008	Shark
MCCL150	*Vibrio harveyi*	Thailand	1990	Shrimp
MCCL151	*Vibrio cincinnatiensisi*	USA	2012	Seawater
MCCL152	*Vibrio harveyi*	USA	1991	seawater
MCCL153	*Vibrio harveyi*	Spain	1990	Sea bass
MCCL154	*Vibrio harveyi*	Spain	1990	Sea bream
MCCL155	*Vibrio harveyi*	Tunisia	1993	Sea bass
MCCL156	*Vibrio harveyi*	Denmark	1993	Shark tank water
MCCL157	*Vibrio harveyi*	Denmark	1993	Shark tank water
MCCL158	*Vibrio harveyi*	Greece	1992	Sea bream
MCCL159	*Vibrio harveyi*	Denmark	2008	Shark
MCCL160	*Vibrio harveyi*	Malta	1993	Sea bream
MCCL161	*Vibrio harveyi*	Denmark	1994	Shark tank water
MCCL162	*Vibrio harveyi*	Italy	1998	Sea bass

MCCL163	*Vibrio harveyi*	Turkey	1997	Sea bass
MCCL164	*Vibrio harveyi*	France	1990	Sea bream
MCCL165	*Vibrio harveyi*	Tunisia	1998	Sea bass
MCCL166	*Vibrio harveyi*	China	1998	Sea perch
MCCL167	*Vibrio harveyi*	China	1996	Sea bass
MCCL168	*Vibrio harveyi*	China	1996	Sea bream
MCCL169	*Vibrio harveyi*	Ecuador	1996	Sea bream
MCCL170	*Vibrio harveyi*	Ecuador	1996	Sea perch
MCCL171	*Vibrio harveyi*	Australia	1990	Seawater
MCCL172	*Vibrio parahaemolyticus*	Japan	2001	Human, Stool
MCCL173	*Vibrio parahaemolyticus*	Thailand	2006	shrimp
MCCL174	*Vibrio parahaemolyticus*	Thailand	2006	shrimp
MCCL175	*Vibrio parahaemolyticus*	Thailand	2006	shrimp
MCCL176	*Vibrio parahaemolyticus*	Thailand	2006	shrimp
MCCL177	*Vibrio parahaemolyticus*	Thailand	2006	shrimp
MCCL178	*Vibrio parahaemolyticus*	Japan	2002	Human, Stool
MCCL179	*Vibrio xuii*	Laizhou,Shadong, China	1995	shrimp culture water
MCCL180	*Vibrio chagasii*	Greece	1991	sea bass
MCCL181	*Vibrio chagasii*	Greece	1991	water
MCCL182	*Vibrio chagasii*	Greece	1991	rotifer
MCCL183	*Vibrio xuii*	Ecuador	2000	white shrimp
MCCL184	*Vibrio chagasii*	Spain	1990	Seawater
MCCL185	*Photobacterium damselae subsp. damselae*	Qingdao, Shandong, China	2007	shrimp
MCCL186	*Vibrio atypicus*	Qingdao, Shandong, China	2007	shrimp
MCCL187	*Photobacterium damselae subsp. damselae*	Qingdao, Shandong, China	2007	shrimp
MCCL188	*Photobacterium damselae subsp. damselae*	Qingdao, Shandong, China	2007	shrimp
MCCL189	*Vibrio alginolyticus*	Qingdao, Shandong, China	2007	shrimp
MCCL190	*Vibrio alginolyticus*	Qingdao, Shandong, China	2007	shrimp
MCCL191	*Vibrio campbellii*	Qingdao, Shandong, China	2007	shrimp
MCCL192	*Vibrio campbellii*	Qingdao, Shandong, China	2007	shrimp
MCCL193	*Vibrio alginolyticus*	Qingdao, Shandong, China	2007	shrimp

MCCL194	*Vibrio alginolyticus*	Qingdao, Shandong, China	2007	shrimp
MCCL195	*Vibrio alginolyticus*	Qingdao, Shandong, China	2007	shrimp
MCCL196	*Vibrio fortis*	Qingdao, Shandong, China	2008	Seawater
MCCL197	*Aggregatibacter actinomycetemcomitans*	Qingdao, Shandong, China	2008	Seawater
MCCL198	*Vibrio harveyi*	Qingdao, Shandong, China	2007	shrimp
MCCL199	*Vibrio splendidus*	Qingdao, Shandong, China	2008	Seawater
MCCL200	*Vibrio rotiferianus*	Yellow Sea, China	2008	Seawater
MCCL201	*Vibrio fortis*	Yellow Sea, China	2008	Seawater
MCCL202	*Vibrio sinaloensis*	Yellow Sea, China	2008	Seawater
MCCL203	*Vibrio neptunius*	Yellow Sea, China	2008	Seawater
